# Citizens' preferences on healthcare expenditure allocation: evidence from Greece

**DOI:** 10.1111/hex.12420

**Published:** 2015-11-02

**Authors:** Sofia Xesfingi, Athanassios Vozikis, Yannis Pollalis

**Affiliations:** ^1^ Department of EconomicsUniversity of PiraeusPiraeusGreece

**Keywords:** citizens' preferences, health expenditure, healthcare allocation, priorities' setting

## Abstract

**Background of context:**

Priority setting and resource allocation across various healthcare functions are critical issues in health policy and strategic decision making. As health resources are limited while there are so many health challenges to resolve, consumers and payers have to make difficult decisions about expenditure allocation.

**Objective:**

Our research focus on the (dis)agreement between citizens' preferences and actual public health expenditure across broad healthcare functions, on whether this (dis)agreement is persistent, on whether various demographic factors amplify this (dis)agreement and to derive useful implications for public health policies.

**Setting and participants:**

Using survey data of 3029 citizens in Greece for the year 2012 and employing logit estimation techniques, we analysed the effect of demographic and other factors in shaping citizens' (dis)agreement with public health expenditure allocation.

**Results:**

Our results demonstrate the important role of income, family members and residence in shaping citizens' preferences regarding health expenditure priorities in almost all healthcare functions, while other demographic factors such as job, age, gender and marital status do partly associate and play a significant role.

**Conclusions:**

Government should encourage the citizens' participation in the decision‐making process in order to eliminate the unveiled and significant disagreement between citizens' preferences and actual public health expenditure across all healthcare functions.

## Introduction

Developed countries spend considerable resources on health, although there are large variations in the levels and rates of growth in the health spending. In 2012, the public spending on health across EU member states was on average 8.7% of their gross domestic product (GDP).^1^ According to recent estimates, spending on health will mount to 20% of GDP by 2050 in most of OECD countries.^2^

Health systems are mostly funded either from general public revenues (e.g. Canada), or through a social security system with a separate budget and hypothecated taxes or contributions (e.g. Australia, France, Belgium, Japan and Germany). Health‐care rationing refers to mechanisms that are used to allocate healthcare resources. As (financial and health services related) resources are limited, to meet health system goals set by the World Health Organization,^3^, ^4^ consumers and payers demand greater accountability and have to make difficult decisions about which health functions to support,^5^ while unequal provision of health services, rapid urbanization and civil conflict are documented, even when the same level of resources is allocated to public health across different countries.^6^ Consequently, priority setting and resource allocation across different health functions are issues of utmost importance for the present and for the years to come.

Although citizens' preferences formation may shape resource allocation decisions in public and private health services delivery, there is still scant evidence on formal public involvement in healthcare priority setting and resource allocation activity.^7^ Early debates on public involvement in healthcare decision making have mainly aimed at strengthening the role of citizens as consumers in the healthcare sector, while later debates emphasized the role of citizen participation and competency as a means of improving the performance of the healthcare system.^8^ Among the recent attempts, the study of Church *et al*.^9^ examined the concept of citizen participation in the context of a series of basic questions through which decision‐makers may draw some policy relevance. This study became a point of reference for an informed discussion of the possibilities for improved citizen participation in healthcare decision making. Whitty *et al*.^10^ discussed the theoretical framework about the optimal approach to access public preferences. Furthermore, Rosen and Karlberg^11^ compared the views of citizens and healthcare decision‐makers on healthcare financing and revealed that the general public have high expectations on public health care that do not fit with the decision‐makers' views on what should be offered. In a review of the empirical literature, Delli Carpini *et al*.^12^ discussed the expectations, drawn from deliberative democratic theory, regarding the benefits (and, for some, pitfalls) assumed to derive from discursive participation and citizen engagement. According to Shaw *et al*.,^13^ citizens require resource allocation decision in health to be informed by considerations of equity as well as efficiency. The study of Dolan and Shaw^14^ demonstrated that people are willing to sacrifice overall health benefits for a more equal distribution of health. Analogous evidence is documented in Schwappach,^15^ where the vast majority of the respondents were willing to trade efficiency for a more equal distribution of resources. In similar vein, the study of Anderson *et al*.^16^ showed that there was strong support among respondents for giving equal priority to people regardless of their personal characteristics, while findings of other studies suggest that health care is informally rationed according to the age and sex of the patient.^17^ Finally, in Wiseman *et al*.^18^ respondents were asked whether they felt the preferences of general public should be used to inform priority setting. Results showed that the public overwhelmingly wanted their preferences to inform priority‐setting decision in health care.

The purpose of this paper was to study whether there is a (dis)agreement between citizens' preferences and actual public spending on a spectrum of healthcare functions, whether this (dis)agreement is persistent across broad healthcare programmes, whether demographic factors of the participants amplify this (dis)agreement and to derive useful implications for public healthcare policies.

We choose to study Greece for three main reasons: first, the out‐of‐pocket health expenditure is higher than anywhere else in the European Union either as a proportion of GDP, or in per capita terms.^19^ Second, the healthcare system in Greece is financed by a mix of public and private resources. Public statutory financing is based on social insurance and tax.^20^ Greece has seen per capita health spending fall by 9% each year since the onset of the severe economic crisis in 2009. Given the tight budgets, it is interesting to analyse the allocation of the limited health resources and whether citizens consent to this.^21^ At the same time, it can be argued that the financial crisis is a no easy way out, as elevated prevalence of certain diseases is already reported,^22^ although many researchers dispute over a causal association between recession and these health outcomes.^23^ Finally, Greece, as also many of the Mediterranean countries, has demographics (low birth rate, high longevity, high unemployment, etc.) that could consist of a serious issue for the future of the healthcare sector.^1^

This paper proceeds as follows: section 2 presents our framework of analysis, data and model. Sections 3 and 4 present and discuss our findings, respectively, and finally, section 5 concludes.

## Methodology

This section presents the research methodology and the data used, and describes the model and the estimation method.

### Data

We conducted a web‐ and interview‐based survey taking a convenient sample^2^ of 3029 persons (citizens) in Greece during the year 2012. Our questionnaire included a wide range of socio‐economic characteristics of the participant citizens, who were requested to allocate a hypothetical amount of money (i.e. €100) in the System of Health Accounts healthcare functions (ICHA‐HC), but including also investment, though treated separately as ‘capital formation’ in health, to meet the total expenditure in health, that is current spending plus capital formation.^24^ Given the actual public spending on all equivalent health programmes, we were able to calculate the size and the statistical significance of the difference between citizens' preferences and the public spending in health care in each healthcare function. Finally, we employed logit estimation techniques to study the effect of demographic factors in shaping citizens' (dis)agreement with public spending on health care.

Table 1, below, presents a short description of these categories.

**Table 1:** The classification of healthcare functions at the first‐digit level

	Health‐care functions	Description
1	HC.1 Curative care	The principal medical intent is to relieve symptoms of illness or injury, to reduce the severity of an illness or injury or to protect against exacerbation and/or complication of an illness which could threaten life
2	HC.2 Rehabilitative care	Emphasis lies on improving the functional levels of the persons served and where the functional limitations are either due to a recent event of illness or injury or of a recurrent nature (regression or progression)
3	HC.3 Long‐term care (health)	On‐going health and nursing care given to inpatients who need assistance on a continuing basis due to chronic impairments and a reduced degree of independence and activities of daily living
4	HC.4 Ancillary services	Clinical laboratory, diagnostic imaging, patient transport and emergency rescue
5	HC.5 Medical goods	Retail trade, fitting, maintaining and renting medical goods and appliances (public pharmacies, opticians, sanitary shops, teleshopping)
6	HC.6 Preventive care	Vaccination campaigns, school health services, prevention of (non)communicable diseases, occupational health care
7	HC.7 Governance, and health system and financing administration	Planning, management, regulation and collection of funds and handling of claims of the delivery system
8	HK Capital account	Capital formation, education and training of health personnel, research and development, environmental health, food and hygiene

*Source*: International Classification of Health Accounts (OECD‐Eurostat‐WHO^24^).

The first five healthcare functions constitute the major component of the personal health services and goods, while functions (6) and (7) form the major component of the public health (collective) services. The sum of functions (1) to (7) constitutes the total current expenditure on health. Finally, adding function (8) one gets the total health expenditure categories.

A number of demographic factors were also requested and recorded from the participants such as Gender, Age, MaritalStatus, Job, Residence, Members and Income. The ordinal variables were classified according to Hellenic Statistical Authority classification standards. More specifically, Gender takes the value of 0 for male and 1 for female; Age consists of six intervals and takes the value of 1 for 15–24, 2 for 25–39, 3 for 40–54, 4 for 55–64, 5 for 65–79 and 6 for >80 years old; MaritalStatus is a categorical variable and takes the value of 1 for singles, 2 for married, 3 for divorcees, 4 for separated and 5 for widows; Job represents the employment status and is 1 for employed, 0 otherwise; Residence indicates the location of residency and is 1 for the prefecture of Athens, 0 otherwise; Members is 1 for a single individual, 2 for a married couple, 3 for a family with one child and so on; Income level is grouped in eight classes and takes the value of 1 for <€750, 2 for €751–1100, 3 for €1101–1450, 4 for €1451–1800, 5 for €1801–2200, 6 for €2201–2800, 7 for €2801–3500, 8 for >€3501.^25^

Table 2, below, presents the summary statistics of our sample participants.

**Table 2:** Descriptive statistics

Variables	Frequency	Percentage	Cumulative percentage
Gender			
Male	1505	46.69	46.69
Female	1524	50.31	100.00
Age			
15–24 years old	519	17.13	17.13
25–39 years old	840	27.23	44.87
40–54 years old	723	23.87	68.74
55–64 years old	347	11.46	80.19
65–79 years old	495	16.34	96.53
>80 years old	105	3.47	100.00
Marital status			
Single	1245	41.10	41.10
Married	1550	51.17	92.27
Divorcee	133	4.39	96.67
Separated	88	2.91	99.57
Widow	13	0.43	100.00
Job			
Unemployed	951	31.40	31.40
Employed	2078	68.60	100.00
Residence			
Other	492	16.24	16.24
Athens	2537	83.76	100.00
Members			
Single individual	737	24.33	24.33
(Married) Couple	458	15.12	39.45
Couple with 1 child	578	19.08	58.53
Couple with 2 children	960	31.69	90.23
Couple with 3 children	255	8.42	98.65
Couple with 4 children	41	1.35	100.00
Income			
≤€750	141	4.66	4.66
€751–1100	404	13.34	17.99
€1101–1450	282	9.31	27.30
€1451–1800	365	12.05	39.35
€1801–2200	348	11.49	50.84
€2201–2800	380	12.55	63.39
€2801–3500	523	17.27	80.65
>€3500	586	19.35	100.00

As Table 2 shows, half of our sample participants are men, while the majority of the participants are between ages of 25 and 39. Participants, on average, are married and have two children. They live in the prefecture of Athens and about 70% of them are employed. Finally, they belong, on average, to middle‐income classes.

Next, Table 3 presents the citizens' preferences to public health expenditure allocation, along with the actual public health spending among healthcare functions in Greece for 2012.

**Table 3:** Summary statistics for health expenditure allocation (citizens' preferences vs. actual public spending)

Variables	Citizens' preferences over health expenditure allocation %					Actual public health expenditure allocation %
	Obs	Mean %	SD	Min %	Max %	
1. Curative care	3029	17.520	8.867	0	60	64.23
2. Rehabilitative care	3029	12.157	6.564	0	75	0.63
3. Long‐term care	3029	11.104	6.337	0	82	0.66
4. Ancillary care	3029	8.633	5.239	0	60	4.01
5. Outpatients	3029	9.095	5.833	0	50	26.55
6. Prevention‐Public health	3029	15.331	8.960	0	79	1.68
7. Administration	3029	11.170	6.521	0	50	2.15
8. Capital formation	3029	14.992	11.078	0	98	0.09

*Source*: A System of Health Accounts: 2001 edition^19^ and own calculations.

Table 3 shows that Greek citizens allocated the hypothetical amount (on health expenditure) almost equally (about 12.5%) across all health categories. Furthermore, they allocated more than half of the budget (almost 60%) to personal health services and goods (variables 1–5), one quarter to collective healthcare services (variables 6–7), and the rest (15%) to capital formation (variable 8). The corresponding actual public expenditure in the aforementioned categories is 64.23, 0.63, 0.66, 4.01, 26.55, 1.68, 2.15 and 0.09, respectively. Information on the public's health expenditure in Greece for the year 2012 among healthcare functions is calculated from OECD data.^19^ As one observes, in health functions (1) and (5), public spending is higher than citizens' preferences, while in the rest of the health functions the opposite holds.

Table 4, below, presents the correlations across healthcare functions (civilian's preferences).

**Table 4:** Correlations across health‐care functions (citizens' preferences)

Variables	1	2	3	4	5	6	7	8
1. Curative care	1.000							
2. Rehabilitative care	0.148^a^	1.000						
3. Long‐term care	−0.068^a^	0.164^a^	1.000					
4. Ancillary care	−0.114^a^	−0.067^a^	0.071^a^	1.000				
5. Outpatients	−0.153^a^	−0.105^a^	−0.098^a^	0.088^a^	1.000			
6. Prevention‐Public health	−0.265^a^	−0.233^a^	−0.214^a^	−0.230^a^	−0.131^a^	1.000		
7. Administration	−0.271^a^	−0.280^a^	−0.213^a^	−0.143^a^	−0.085^a^	0.067^a^	1.000	
8. Capital formation	−0.340^a^	−0.364^a^	−0.298^a^	−0.159^a^	−0.171^a^	−0.198^a^	−0.026	1.000

a Significance at 5% level of significance.

As Table 4 shows, there is no strong correlation across healthcare functions, as the Pearson correlation coefficient is small (smaller than 0.3 in most cases). A stronger association, however, is demonstrated between the variables ‘capital formation’ with ‘curative care’ (0.34) and ‘rehabilitative care’ (0.36).

So far, we have discussed how citizens have expressed their preferences for allocating a hypothetical amount of money (budget) across major healthcare functions. This allocation reveals only the preferences of the citizens on how the government should allocate (and prioritize) the expenditure across these healthcare functions. Nevertheless, actual public health expenditure on these functions seems to be indeed very different.

To statistically examine these differences, we performed the following test: the citizens' expenditure allocation preferences means were tested under the hypothesis that they are equal with the public health expenditure allocation means in every healthcare function (variable). We reject the null hypothesis at 95% interval confidence (*a* = 5% level of significance) for all cases. Therefore, the means of the citizens' preferences are statistically different from the actual public expenditure means for all eight functions. Consequently, there seems to be some disagreement between citizens' preferences and actual public expenditure on health expenditure allocation.

Furthermore, our study aimed to quantify this ‘disagreement’. In doing so, we took the difference between the two stakeholders' (citizens and government) means, for each of the eight variables (functions), and calculated the distribution of deviations. Then, we introduce a dummy variable taking the value of 1 for ‘strong’ disagreement between the two stakeholders for deviations higher than the 66th percentile of the distribution; 2 for ‘modest’ agreement for deviations between the 3rd and 66th percentile of distribution; and finally, 3 for ‘almost’ agreement for deviations below the 33rd percentile of the distribution.

In the next section, we present our model, which aims to explain the sources of this (dis)agreement.

### Model

The likelihood of a citizen's preferences to coincide with actual public health expenditure allocation can be described by an ordered logit model as follows:

$$\text{Pr}\left(Y=c|X_{i}\right)=F\left(X_{i}\beta \right),$$

where the endogenous variable *Y* is the degree of citizens' agreement with actual public health expenditure allocation and is an integer ranging from 1 (fully disagree) to 3 (fully agree); *F* is the standard logistic cumulative distribution function; and *Χ* is a set of covariates detailed below. The model is specified as:

$$\begin{array}{cc} Y_{i}= & \beta_{0}+\beta_{1}{\text{Gender}}_{i}+\beta_{2}{\text{Age}}_{i}\\ & +\beta_{3}{\text{MaritalStatus}}_{i}+\beta_{4}{\text{Job}}_{i}\\ & +\beta_{5}{\text{Residence}}_{i}+\beta_{6}{\text{Members}}_{i}\\ & +\beta_{7}{\text{Income}}_{i}+\varepsilon_{i},\varepsilon_{i}∼\text{Logistic}\left(0,1\right) \end{array}$$

where Gender is a dummy variable that takes the values 0 and 1 if the citizen is male and female, respectively; Age is the age of the citizen and is a dummy that takes the value of 1 (for ages 15–24), 2 (for ages 25–39), 3 (for ages 40–54), 4 (for ages 55–64), 5 (for ages 65–79) and 6 (for ages >80 years old); MaritalStatus is a dummy and is 1 for singles, 2 for married, 3 for divorced, 4 for separated and 5 for window; Job is a dummy for the employment status of the citizen and takes the values 0 for unemployed and 1 employed; City is a dummy variable that takes the value 1 if the citizen lives in Athens and 0 otherwise; Members is the citizen's total family members (is 1 for a single person, 2 for a married couple, 3 for a family with one child and so on; Income is a dummy for the income level of the citizen and is 1 for income level <€750, 2 for €751–€1100, 3 for €1101–€1450, 4 for €1451–€1800, 5 for €1801–€2200, 6 for €2201–€2800, 7 for €2801–€3500 and 8 for income level > €3501.

The selection of the variables in *Χ*_i_ set can be justified by various studies.^3^, ^11^, ^16^, ^26^, ^27^, ^28^, ^29^, ^30^ More particularly, Anderson *et al*.^16^ identified five key clusters of factors that contribute to explaining the healthcare preferences of the general public. These factors are as follows: age, marital status, educational level, social welfare and general religiosity.

## Results

Table 5, below, presents estimates of odds ratios for each one of the eight healthcare functions. One can read the odds ratios as follows: if the odd ratio, *a*, is bigger than 1 (*a* > 1), then the probability of a citizen being satisfied with the actual public health expenditure allocation, that is *Y* = 3 (full agreement), increases by (*a*–1) × 100%, whereas the probability decreases by (1–*a*) × 100%, if the odd ratio is smaller than one (*a* < 1).

**Table 5:** Logit estimates (odds ratios) for various health‐care functions (dependent variable: Deviation between citizens' preferences and actual public health expenditure)

Odds ratios	Personal health services and goods					Public (Collective) health‐care services		Capital formation
	1	2	3	4	5	6	7	8
	Curative care	Rehabilitative care	Long‐term care	Ancillary care	Outpatients	Prevention‐Public Health	Administration	Capital formation
Gender	0.975 (0.191)	1.231*** (0.094)	0.939 (0.078)	0.937 (0.148)	1.041 (0.106)	1.008 (0.075)	0.852* (0.083)	1.194** (0.085)
Age	0.875 (0.073)	0.932* (0.031)	1.026 (0.037)	1.067 (0.077)	0.936 (0.043)	0.976 (0.032)	0.980 (0.044)	1.091*** (0.034)
MaritalStatus	1.062 (0.135)	1.007 (0.059)	0.851** (0.055)	1.105 (0.165)	0.989 (0.090)	0.997 (0.062)	1.191** (0.101)	1.010 (0.057)
Job	3.322*** (0.943)	0.974 (0.090)	1.246** (0.124)	1.260 (0.227)	0.884 (0.105)	0.917 (0.082)	0.758** (0.092)	1.389*** (0.119)
Residence	0.610** (0.140)	0.748*** (0.080)	0.903 (0.104)	1.344 (0.264)	0.693*** (0.089)	0.756*** (0.082)	1.152 (0.147)	1.180* (0.115)
Members	1.041 (0.076)	0.917*** (0.029)	0.910*** (0.030)	1.008 (0.066)	1.146*** (0.049)	1.170*** (0.036)	1.094** (0.045)	0.974 (0.030)
Income	0.783*** (0.039)	1.037* (0.020)	1.061*** (0.022)	1.051 (0.041)	0.937** (0.024)	0.932*** (0.017)	1.007 (0.025)	0.998 (0.018)
Pseudo‐*R* ^2^	0.0614	0.0057	0.0072	0.0072	0.0117	0.0081	0.0075	0.0055
Wald	50.13	26.45	29.40	12.85	34.25	43.02	23.72	31.22
Obs	3029	3029	3029	3029	3029	3029	3029	3029

*Note*. Heteroscedasticity‐robust standard errors in parentheses.

***, **, * indicate significance at 1, 5 and 10%, respectively.

According to Table 5, all demographic factors explain the deviations between citizens' preferences with actual public health expenditure allocation. Among the demographic factors, Members, Residence and Income appear to be statistically significant in the majority (5 of 8) of healthcare functions. The factor Job is statistically significant in almost half of the healthcare functions, Gender is statistically significant in three functions, and finally, Age and MaritalStatus are statistically significant in only two functions.

More specifically, the number of family members (Members) has a positive and statistically significant role in the majority of healthcare functions. For instance, for the category ‘medical goods and services dispensed on outpatients’ (5), when an additional member enters in a participant's family, the probability of a citizen's preference to be in agreement with public health expenditure allocation increases by 14.6% [=(1.146–1) × 100%]. Similar positive effect is also documented for the functions ‘public health‐prevention’ (6) and ‘administration’ (7), where the probability of a citizen to be satisfied with public health expenditure allocation increases by 17 and 9.4%, respectively. However, the opposite holds for the categories of ‘rehabilitative care’ (2) and ‘long‐term care’ (3). In particular, when a citizen's family is getting bigger, then his/her probability of being satisfied with public health expenditure allocation decreases by 8 and 9%, respectively.

Furthermore, the income class of a participant (Income) has a positive and statistically significant association with the function ‘long‐term care’ (3). As the citizen's level of income increases and changes income class, the probability of being satisfied increases by 6.1%. For the functions ‘curative care’ (1), ‘medical goods dispensed to outpatients’ (5) and ‘prevention‐public health’ (6), the income effect is negative. That means the higher the level of income of a citizen is, the probability of being in agreement with public's spending decreases by 21.7, 6.3 and 6.8%, respectively.

Where the civilian resides (Residence) also plays a role in a civilian's preferences and perception of health rationing. This factor is statistically associated with the health categories of ‘curative care’ (column 1), ‘rehabilitative care’ (column 2), ‘medical goods and services dispensed to outpatients’ (column 5), ‘prevention‐public health’ (6) and ‘capital formation’ (column 8). In the latter case, there is a positive association, with the probability of a civilian being in fully agreement with public health expenditure allocation to increase by 18% if the citizen moves from the rest of the country to the prefecture of Athens. In all other aforementioned cases, the Residence effect is negative and the average decrease of a citizen's probability of being in fully agreement with the actual public health expenditure allocation is 30%.

The employment status of a citizen is also an important factor for shaping the degree of (dis)agreement between public and his/her own hypothetical expenditure allocation. The estimate of Job is statistical significant in four functions, namely ‘curative care’ (1), ‘long‐term care’ (3), ‘administration’ (7) and ‘capital formation’ (8). More specifically, there is a positive association with respect to ‘curative care’. Positive is also the Job effect for the functions ‘long‐term care’ (3) and ‘capital formation’ (8). When a citizen is employed, the probability of being in fully agreement with the actual public health expenditure allocation increases by 24.6 and 38.9%, respectively, compared to an unemployed person. The opposite effect is documented for the ‘administration’, where the holding of a job leads to a decrease of the probability of in fully agreement by 24.2%.

The factor Gender seems to be statistically important only for the function ‘rehabilitative care’ (2) and ‘capital formation’ (8). In both variables, there is a positive and strong effect (at 1 and 5% level of significance, respectively), while a negative but with marginal statistical significance (at 10% level of significance) is documented for the function ‘administration’ (7). More particularly, women are more likely to be in agreement with actual public health expenditure allocation [about 23.1 and 19.6%, respectively, for the functions (2) and (8)] compared to a man.

Further, the demographic factor of Age seems to be statistically significant only for the function ‘capital formation’ (8). We find that as the citizens grow older, the likelihood of being in fully agreement with actual public health expenditure allocation increases by 9.1%. A marginal significance is also demonstrated for the function ‘rehabilitative care’ (2).

Finally, the marital status (MaritalStatus), which plays an important role in two functions that of ‘long‐term care’ (3) and ‘capital formation’ (8), is a categorical variable, that is there is no intrinsic ordering to the categories, and, therefore, a marginal effect analysis is required and performed in Table 6 in this section below.

**Table 6:** Marginal effects analysis

Marginal effect	Long‐term care	Administration
MaritalStatus		
1 (single)	0.747 (0.015)	0.795 (0.014)
2 (married)	0.718 (0.013)	0.843 (0.010)
3 (divorced)	0.667 (0.043)	0.795 (0.039)
4 (separated)	0.620 (0.052)	0.886 (0.033)
5 (widow)	0.842 (0.100)	0.772 (0.133)

*Note*. Heteroscedasticity‐robust standard errors in parentheses.

Table 6, below, presents the marginal effect analysis for MaritalStatus and for the functions in which appear to be statistically significant.

The marginal effect analysis of the marital status effect can be read as follows: the probability of a citizen being satisfied because the government met his/her preferences with respect to public health expenditure of ‘long‐term care’ function is 74.7% among those who are single, 71.8% among those who are married, 66.7% among those who are divorced, 62% among those who are separated and 84.2% among widowers. With respect to the function ‘administration’, the probabilities are 79.5, 84.3, 79.5, 88.6 and 77.2%, respectively.

## Discussion

Ageing population, shifting demographics, rising unemployment and financial strain, increasing healthcare costs and reductions in tax revenues are contributing to deeply stress the Greek healthcare system, while decreased disposable income has made access to health care more difficult for many households.^31^ The citizens' extremely low level of satisfaction from the Greek Health System^32^ reflects the impact of economic crisis and austerity in health care and in the social policy in general.^30^

In this context, policymakers and service providers are faced with the challenge of better allocate the available (scarce) resources. Priority setting and better allocation in healthcare expenditure are being introduced as a means to overcome these problems and to provide a fair distribution of resources.^11^ Health‐care expenditure is both determined exogenously, through non‐system external pressures, which may occur at the macroeconomic level, and endogenously, through factors that impact directly on expenditure and are determined mostly at the microeconomic level through a complex set of relationships.^33^ A common approach to policy formulation in the face of resource constraints is to adopt the framework of societal health benefits maximization through reliance on the cost‐effectiveness of health services provision, though does not always seem to be socially accepted.^34^ On the other hand, the Accountability for Reasonableness (A4R) framework^35^, ^36^ states that power differences must be mitigated to facilitate effective participation of diverse members in the decision‐making context for priority setting in healthcare fin‐ancing. Finally, Botelho *et al*.^37^ found that although citizens wish to be consulted, they believe doctors should play the most important role on health expenditure allocation and rationing decisions.

In our research, we found that the number of family members seems to play a significant role shaping the citizens' agreement with respect to actual public health expenditure allocation, in the majority of healthcare functions. The effect, however, of this demographic factor, is not the same in all cases. ‘Collective health services’, for example, have a great impact on children, as vaccination is essential. The same is true with the ‘medical goods and services dispensed to outpatients’ function as it includes public pharmacies and sanitary shops. In contrast, the ‘long‐term care’ and ‘rehabilitative care’ are not highly ranked in parents' preferences, finding present in other study for Greece.^38^

Other demographic factors such as job, age, gender and marital status do partly associate and play a significant role. These findings are consistent with other studies where these criteria for prioritizing medical services have also controversial results.^27^, ^28^, ^33^, ^39^, ^40^ However, other findings^38^, ^41^, ^42^ indicate that personal characteristics such as gender, age, education are context specific of choices in health.

With respect to income, it appears that poor citizens in Greece are in agreement with the public spending in the biggest health functions which are ‘curative services’ and ‘medical goods and services dispensed to outpatients’. The importance of income in ‘collective health services’ is also reasonable.^26^ The higher the income class of a citizen, the lower his/her hypothetical spending on this function will be. Civilians would prefer more expenditure to be allocated to the functions of ‘curative care’ and to ‘medical goods and services dispensed to outpatients.’ This is also quite reasonable as these functions are very important in daily life, in contrast to the ‘long‐term nursing care’ function, which usually include chronic impairment. Citizens tend to focus more on present needs and less on future or expected chronic situations.^28^, ^43^

The preferences of citizens, who live outside the prefecture of Athens, seem to be in disagreement with actual public health expenditure for the majority of healthcare functions. We must not forget that mechanisms for needs assessment and priority‐setting are underdeveloped in the Greek Health System and, as a consequence, the regional distribution of health resources is unequal.^20^ Our estimate on the variable Capital formation, which shows that the citizens tend to agree with that reality, is consistent with similar findings as appear in the Coelho^44^ study.

In addition, one would expect an employed civilian to allocate more resources to all categories that potential directly related to his/her medical treatment and the utility s/he drives currently or in the future for the medical system and its functions.^45^ Such health services are those of ‘curative care’, which is covered by his/her insurance, or ‘long‐term nursing care’, which may cover the possibility of a labour accident, whereas spending on the category ‘regulation and collection of funds’ would not rank high in his/her preferences.^29^, ^46^

Overall, our results demonstrate that there is a large deviation between citizens’ preferences and actual public spending with respect to the resources allocated particularly to the functions of ‘curative care services’ (strong disagreement) and ‘medical goods and services dispensed to outpatients’ (modest disagreement).^3^ In all other health functions, the deviation documented is relatively small, thus we consider that public spending almost meets citizens’ preferences.

Demographic factors seem to play an important role in explaining the deviations between citizens’ preferences and public health spending. More particularly, our findings show the important role of income, family members and residence in shaping citizens’ preferences regarding health financing priorities in almost all healthcare functions, while other demographic factors such as job, age, gender and marital status do partly associate and play a significant role.

Several studies demonstrated so far that there is a gap between public preferences and actual public spending on health care. According to Rosen and Karlberg,^11^ the general public have high expectations of public care, expectation that do not fit with the decision‐makers’ views on what it should be offered. Nevertheless, the majority of these studies first focus on how the citizens rank different population groups in terms of their importance, and second on how the citizens’ involvement in the decision‐making process would lead in a more effective allocation. To our knowledge, this is the first attempt in the literature that the citizens’ preferences are studied in terms of healthcare functions’ funding; therefore, we are not able to perform comparisons with existed related studies.

Finally, it is worth to further evaluate the construction of the corresponding questionnaire as there was no methodology to base upon. Although it is demonstrated that income, the numbers of family members and residence play an important role in shaping citizens’ preferences, further research is needed to be carried out in order to evaluate the potential effect of confounding factors. Our methodology for the construction of the citizens’ agreement index is based first on the sample average and second on the comparison of each healthcare function funding with respect to the aforementioned average. We also experimented with alternative indices constructed with higher deviations with respect to the proposed one and results do not change significantly. So we may assume that even with the addition of some data, a deviation between citizens’ preferences and actual public spending on health will still be presented.

## Conclusions

Government and citizens’ rankings alongside health are one of the general topics they are most interested in. But still there are wide disparities between the level and the means of participation in the decision‐making process. Priority setting and resource allocation across various healthcare functions are critical issues in health policy and strategic decision making. As health resources are limited while there are so many health challenges to resolve, consumers and payers have to make difficult decisions about expenditure allocation.

Our research unveiled the significant disagreement between citizens’ preferences and actual public health expenditure across all healthcare functions, focusing on various demographic factors and deriving useful implications for public health policies.

As a result, government should encourage the citizens’ participation, by introducing policies of empowering the knowledge dissemination and democratization in the decision‐making process.

## Conflict of interest

No conflict of interest declared.

## Notes

1 According to OECD^1^ the population age structure in Greece is very similar to other Mediterranean countries, especially Italy and Portugal (almost 20% of total population is above the age of 65 years old). Although life expectancy is the same in all those countries, the percentage of permanent employment in Greece is the lowest (34.2%) and at the same time the percentage of long‐term unemployment is the highest (from 45% for the year 2011, jumped to 55.6 and 66.4% for the years 2012 and 2013, respectively).

2 Convenience sampling is a non‐probability sampling technique where subjects are selected just because of their convenient accessibility and proximity to the researcher; therefore, the subjects are selected just because they are easiest to recruit for the study.

3 These two particular health functions enjoy the biggest proportion of public spending in Greece and elsewhere in the Mediterranean countries (e.g. Italy, Portugal and Spain). However, in Greece, public funding of ‘medical goods and services dispensed to outpatients’ is almost double or triple compared to for instance Spain and Italy/Portugal, respectively.
